# Origin and Roles of Alanine and Glutamine in Gluconeogenesis in the Liver, Kidneys, and Small Intestine under Physiological and Pathological Conditions

**DOI:** 10.3390/ijms25137037

**Published:** 2024-06-27

**Authors:** Milan Holeček

**Affiliations:** Department of Physiology, Faculty of Medicine, Charles University, 500 03 Hradec Kralove, Czech Republic; holecek@lfhk.cuni.cz

**Keywords:** glucose, branched-chain amino acids, diabetes, starvation, cirrhosis

## Abstract

Alanine and glutamine are the principal glucogenic amino acids. Most originate from muscles, where branched-chain amino acids (valine, leucine, and isoleucine) are nitrogen donors and, under exceptional circumstances, a source of carbons for glutamate synthesis. Glutamate is a nitrogen source for alanine synthesis from pyruvate and a substrate for glutamine synthesis by glutamine synthetase. The following differences between alanine and glutamine, which can play a role in their use in gluconeogenesis, are shown: (i) glutamine appearance in circulation is higher than that of alanine; (ii) the conversion to oxaloacetate, the starting substance for glucose synthesis, is an ATP-consuming reaction for alanine, which is energetically beneficial for glutamine; (iii) most alanine carbons, but not glutamine carbons, originate from glucose; and (iv) glutamine acts a substrate for gluconeogenesis in the liver, kidneys, and intestine, whereas alanine does so only in the liver. Alanine plays a significant role during early starvation, exposure to high-fat and high-protein diets, and diabetes. Glutamine plays a dominant role in gluconeogenesis in prolonged starvation, acidosis, liver cirrhosis, and severe illnesses like sepsis and acts as a substrate for alanine synthesis in the small intestine. Interactions among muscles and the liver, kidneys, and intestine ensuring optimal alanine and glutamine supply for gluconeogenesis are suggested.

## 1. Introduction

Gluconeogenesis is a metabolic pathway of glucose synthesis from non-carbohydrate substances essential for maintaining glycemia during starvation, prolonged exercise, and a carbohydrate-deficient diet. It contributes to increased glucose levels in diabetes and disorders associated with insulin resistance. The primary substrates are lactate, glycerol, and glucogenic amino acids. Glucogenic amino acids are mostly those that can be converted to oxaloacetate, a substance that initiates gluconeogenesis in a reaction catalyzed by phosphoenolpyruvate carboxykinase (PEPCK). Gluconeogenesis is regulated by several translational and posttranslational mechanisms [1,2,3,4]. In most conditions, but not all, gluconeogenesis is stimulated synergistically by increasing the supply of substrates and catabolic hormones, particularly glucagon, catecholamines, and cortisol.

It has been believed for many years that alanine is the primary glucogenic amino acid and that the liver has a central position in gluconeogenesis [5,6]. However, this idea has been challenged by reports demonstrating that glutamine and the kidneys have much more significance in glucose homeostasis than was expected [3,7,8,9,10] and suggesting that because the main substrate for alanine synthesis is pyruvate formed during glycolysis, alanine should instead be considered as a substance that ensures glucose recycling between the liver and muscles via the “alanine cycle,” rather than a substrate for gluconeogenesis [11,12,13]. Although there are many data in the scientific literature, the roles of alanine and the liver on one side and glutamine and kidneys on the other in maintaining glucose homeostasis are not yet fully understood. Furthermore, several lines of evidence support the view that gluconeogenesis also occurs in the small intestine [4,14,15].

This study aimed to explain the pathways of endogenous production of alanine and glutamine and clarify their roles in gluconeogenesis in the liver, kidneys, and small intestine under various physiological and pathological conditions, including starvation, high-protein and high-fat diets, diabetes, and liver cirrhosis.

## 2. Sources of Alanine and Glutamine in the Body

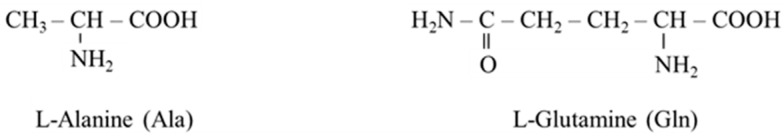



Alanine and glutamine are quantitatively the most important glucogenic amino acids due to their simple conversion to oxaloacetate and much higher concentrations and appearance rates in plasma compared to other glucogenic amino acids. Alanine concentrations (~0.3 mM) and glutamine (~0.6 mM) comprise more than 60% of the free α-amino acid pool in plasma. In healthy humans in a postabsorptive state, plasma alanine’s appearance rate is ~200 µmol/kg per h (~30 g per day) and ~325 µmol/kg per h (~80 g per day) for glutamine [16], which is several times higher than the appearance rates of other amino acids and approximately ten times higher than daily intake of alanine and glutamine in food.

### 2.1. Alanine and Glutamine Synthesis in Skeletal Muscle

**Figure 1:**
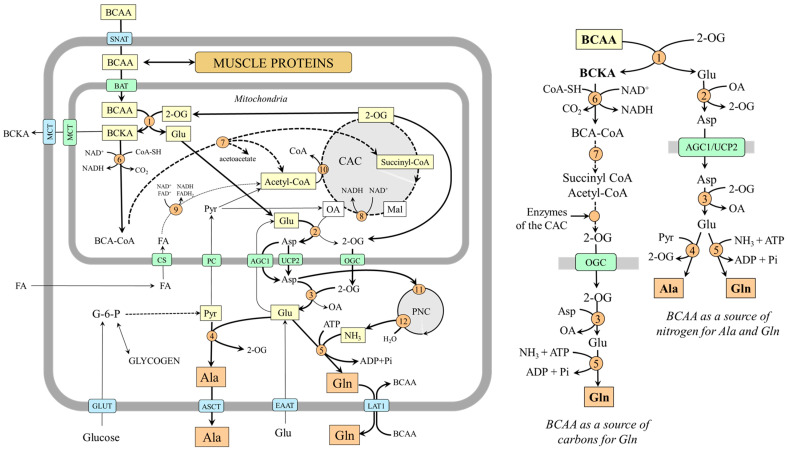
BCAAs as a substrate for alanine and glutamine synthesis in muscles. 1, BCAA aminotransferase; 2, AST (mitochondrial); 3, AST (cytosolic); 4, ALT; 5, glutamine synthetase; 6, BCKA dehydrogenase; 7, enzymes converting BCA-CoAs into acetyl-CoA, acetoacetate, or succinyl-CoA; 8, malate dehydrogenase (mitochondrial); 9, β-oxidation; 10, citrate synthase; 11, adenylosuccinate synthetase; 12, adenylate deaminase. Abbreviations: AGC1, aspartate–glutamate carrier; ASCT, alanine, serine, cysteine, and threonine carrier; BAT, BCAA transporter (SLC25A4); BCAA, branched-chain amino acids; BCA-CoA, branched-chain acyl-CoA; BCKA, branched-chain keto acids; CAC, citric acid cycle; CS, carnitine system; EAAT, excitatory amino acid transporters; GLUT, glucose transporter; LAT1, large neutral amino acid transporter 1; MCT, monocarboxylate transporter; OA, oxaloacetate; OGC, 2-oxoglutarate carrier; PC, pyruvate carrier; PNC, purine-nucleotide cycle; Pyr, pyruvate; SNAT, sodium neutral amino acid transporter; 2-OG, 2-oxoglutarate.

Early studies have demonstrated that the main source of alanine and glutamine in the body is their endogenous synthesis in skeletal muscle and that branched-chain amino acids (BCAAs; valine, leucine, and isoleucine) are a donor of amino nitrogen to 2-oxoglutarate (2-OG) to form glutamate, which is converted by aspartate aminotransferase (AST) to aspartate in the mitochondria [17,18,19,20]. Aspartate is transported to the cytosol via uncoupling protein 2 (UCP2) or aspartate–glutamate carrier 1 (AGC1, aralar 1), a component of the malate–aspartate shuttle [21,22]. In the cytosol, aspartate provides its amino group for the synthesis of glutamate, which acts as the main source of nitrogen for alanine synthesis by alanine aminotransferase (ALT) and a substrate for glutamine synthesis by glutamine synthetase [17]. The primary source of pyruvate for alanine synthesis is glycolysis, and the source of ammonia for glutamine synthesis is the purine–nucleotide cycle [23]. Because aspartate can enter the purine–nucleotide cycle, its amino group originating from the BCAA can also appear as an amide group in glutamine (Figure 1). Alanine is released from muscles through several transporters including ASCT1 and ASCT2, and glutamine is released mainly by LAT1 [24].

BCAA transamination and the production of glutamine and alanine are activated under the condition of increased BCAA supply to BCAA aminotransferase, for instance, with a protein-rich diet, in the initial phase of starvation, during exercise, and in muscle wasting disorders [17]. The ratio between the amount of alanine and glutamine released into the bloodstream depends on the supply of pyruvate and ammonia. If glycolysis is active, alanine is preferentially synthesized and released from muscles, for instance, during fasting, when glycolysis and glycogenolysis are activated in the muscles. In hyperammonemia, e.g., due to liver injury, glutamine synthetase is activated, and glutamine becomes the main product.

### 2.2. BCAAs Can Be a Source of Carbons for Glutamine sSynthesis

BCAAs can become a source of carbons for glutamine synthesis when branched-chain keto acids (BCKAs) produced in muscles by BCAA aminotransferase are not released into the circulation but oxidized by BCKA dehydrogenase, the rate-limiting enzyme of BCAA catabolism. The branched-chain acyl-CoAs produced by BCKA dehydrogenase are degraded gradually into acetyl-CoA and succinyl-CoA, which can enter the CAC, and their carbons appear in 2-OG, which can be subsequently converted to glutamine via transamination and amidation reactions. The isotopic tracing technique has confirmed the possibility. Incubation of muscles with [U-^14^C]BCAA, [U-^14^C]valine, or [U-^14^C]BCKA demonstrated that the carbons appear in glutamine and glutamate but not alanine [25,26]. The role of the CAC in glutamine synthesis has been shown using incubation of rat diaphragms with [2,3-^14^C]succinate in which the ^14^C recovered from alanine was only 2 or 3% of that in glutamine and glutamate [25].

It should be noted that succinate originating from valine or isoleucine can provide four carbons to make a 2-OG molecule, whereas only one carbon can originate from acetyl-CoA produced by leucine or isoleucine catabolism. Acetyl-CoA provides two carbons to oxaloacetate to form citrate (contains six carbons) that is gradually converted to 2-OG made up of five carbons, of which only one is from acetyl-CoA. Assuming simultaneous utilization of all three BCAA, all 2-OG carbons can be derived from BCAAs.

Because BCKA dehydrogenase activity in muscles is very low, BCAAs can become a source of carbon for glutamine synthesis only in certain metabolic conditions in which BCKA dehydrogenase is activated. For instance, in the final stage of starvation, untreated diabetes, severe injury, and sepsis [27,28,29,30].

### 2.3. Alanine and Glutamine Synthesis in Other Tissues

In addition to muscles, alanine and glutamine can be released from adipose tissue and lungs, glutamine from the brain and liver, and alanine from the small intestine and kidneys. In adipose tissue, alanine and glutamine synthesis is associated with using BCAAs for fat synthesis and stimulated by insulin in a postprandial state [31,32]. In the lungs, glutamine synthetase expression and glutamine production are increased in post-operative conditions, trauma, and sepsis [33]. The main site of glutamine synthesis in the brain is astrocytes [34]. The pathways of glutamine synthesis in the liver by perivenous hepatocytes and alanine synthesis in the small intestine and kidneys are described in the following sections.

## 3. Alanine and Glutamine Conversion to Oxaloacetate

**Figure 2:**
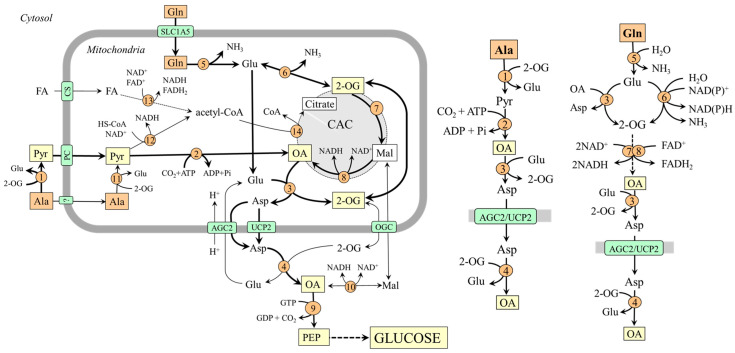
Pathways of alanine and glutamine conversion to oxaloacetate in mitochondria and its delivery to the cytosol in the form of aspartate. 1, ALT (cytosolic); 2, pyruvate carboxylase; 3, AST (mitochondrial); 4, AST (cytosolic); 5, glutaminase; 6, GDH1(2); 7, enzymes converting 2-OG to malate; 8, malate dehydrogenase (mitochondrial); 9, PEPCK; 10, malate dehydrogenase (cytosolic); 11, ALT (mitochondrial); 12, pyruvate dehydrogenase; 13, 3-hydroxyacyl-CoA dehydrogenase; 14, citrate synthase. Abbreviations: AGC2, aspartate–glutamate carrier; CAC, citric acid cycle; CS, carnitine system; FA, fatty acids; Mal, malate; OA, oxaloacetate; OGC, 2-oxoglutarate carrier; PC, pyruvate carrier; PEP, phosphoenolpyruvate; Pyr, pyruvate; UCP2, uncoupling protein 2; 2-OG, 2-oxoglutarate.

The pathways of alanine and glutamine conversion to oxaloacetate, the starting substance of gluconeogenesis, differ and can be influenced by various factors (Figure 2).

### 3.1. Oxaloacetate Synthesis from Alanine

Alanine is converted to oxaloacetate in two steps catalyzed by ALT and pyruvate carboxylase:

Ala + 2-OG ↔ Pyr + Glu

Pyr + CO_2_ + ATP → OA + ADP + Pi

The highest ALT activities are in the liver, small intestine, muscles, heart, and kidneys [35]. However, a pronounced flux toward pyruvate is only in the liver. High levels of pyruvate carboxylase are in the liver and kidney cortex, lower in other tissues, including the small intestine [36].

For the fate of pyruvate, it is significant that pyruvate carboxylase is allosterically activated by acetyl-CoA and requires ATP, whereas pyruvate dehydrogenase, which ensures pyruvate conversion to acetyl-CoA, is inhibited by NADH. Therefore, pyruvate conversion to oxaloacetate by pyruvate carboxylase will be prioritized over the flux through pyruvate dehydrogenase under conditions of increased β-oxidation due to enhanced supply of acetyl-CoA, NADH, and ATP. This means that gluconeogenesis from pyruvate can be facilitated.

### 3.2. Oxaloacetate Synthesis from Glutamine

Glutamine is converted to oxaloacetate in three steps catalyzed by glutaminase, glutamate dehydrogenase (GDH) or mitochondrial AST, and enzymes of the CAC:
GlutaminaseGln + H_2_O → Glu + NH_3_GDH or AST Glu + H_2_O + NAD(P)^+^ ↔ NH_3_ + 2-OG + NAD(P)H + H^+^Glu + OA ↔ Asp + 2-OG2-OG → OA (CAC)2-OG + NAD^+^ + FAD^+^ + H_2_O ↔ **OA** + NADH+H^+^+ FADH_2_ + CO_2_


Glutaminase exists in two isoforms–hepatic and renal. The hepatic type is expressed in periportal hepatocytes, the brain, and the pancreas. The renal type is abundant in kidneys, brain, intestine, and leucocytes. A drop in pH inhibits the liver type, whereas the renal type is activated [37]. GDH, like glutaminase, also exists in two forms. GDH1, located in the liver, can both remove and release ammonia [38]. GDH2, which shows a lower optimal pH, ensures the flux towards ammonia in the proximal tubules of the kidneys [39]. The flux through mitochondrial AST is driven toward the direction of 2-OG by aspartate–glutamate carrier 1/2 (AGC1/2), which continuously supplies glutamate and protons into mitochondria and removes aspartate. AGC2 (citrin) is strongly expressed in all three gluconeogenic organs [40].

The conversion of 2-OG to oxaloacetate through the CAC is ensured by oxoglutarate dehydrogenase, succinate dehydrogenase, fumarase, and malate dehydrogenase and is associated with the synthesis of NADH and FADH_2_. Therefore, (i) the conversion of glutamine to oxaloacetate can result in an increased ATP formation, and (ii) the flux of 2-OG toward oxaloacetate via the CAC can be inhibited by the increased supply of NADH and FADH_2_ from other sources, for instance, due to enhanced oxidation of fatty acids after increased dietary intake of lipids.

### 3.3. Oxaloacetate Transport from the Mitochondria into the Cytosol

Because the inner mitochondrial membrane is impermeable to oxaloacetate, a special form of oxaloacetate delivery into the cytosol, where the enzymes of gluconeogenesis are located, is required. The main is probably the form of aspartate synthesized by the mitochondrial AST (OA + Glu → Asp + 2-OG) and transported into the cytosol through AGC2 or UCP2 [21,40]. In the cytosol, aspartate is converted to oxaloacetate by the cytosolic form of AST (Asp + 2-OG → OA + Glu). Transport in the form of malate, an option noted in textbooks of biochemistry, is less likely [41].

## 4. Gluconeogenesis from Alanine and Glutamine in Liver, Kidneys, and Small Intestine

Most studies on the use of alanine and glutamine for gluconeogenesis conclude that alanine is the primary amino acid for gluconeogenesis in the liver, whereas glutamine plays the leading role in kidneys and small intestine [4,8,42,43].

### 4.1. Gluconeogenesis from Alanine and Glutamine in the Liver

In the liver, the enzymes of gluconeogenesis are expressed together with enzymes of the urea cycle in periportal hepatocytes (the peripheral part of the liver lobule), and it is consensus that hepatic gluconeogenesis from amino acids is associated with urea synthesis [44,45,46].

**Figure 3:**
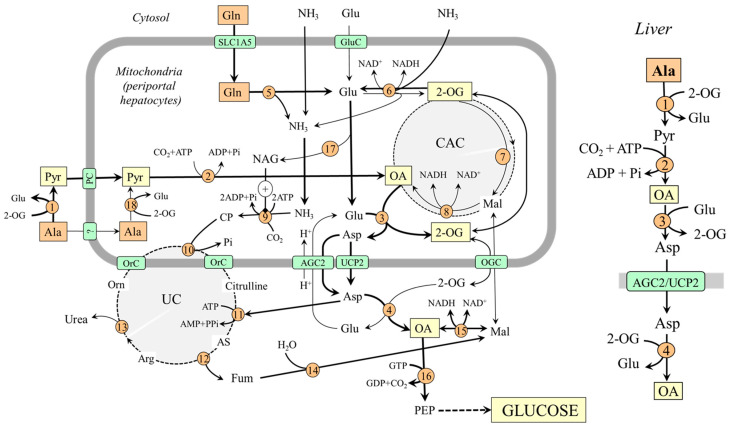
Gluconeogenesis from alanine and glutamine in the liver and its relationship with urea synthesis. On the right is depicted the pathway of oxaloacetate synthesis from alanine; the pathway from glutamine is less significant under usual conditions. 1, ALT (cytosolic); 2, pyruvate carboxylase; 3, AST (mitochondrial); 4, AST (cytosolic); 5, glutaminase; 6, GDH1; 7, enzymes converting 2-OG to malate; 8, malate dehydrogenase (mitochondrial); 9, carbamoyl phosphate synthetase; 10, ornithine carbamoyltransferase; 11, argininosuccinate synthetase; 12, argininosuccinate lyase; 13, arginase; 14, fumarase; 15, malate dehydrogenase (cytosolic); 16, PEPCK; 17, N-acetylglutamate synthase; 18, ALT (mitochondrial). Abbreviations: AGC2, aspartate–glutamate carrier 2; AS, argininosuccinate; CAC, citric acid cycle; CP, carbamoyl phosphate; GluC, glutamate carrier; Mal, malate; NAG, N-acetylglutamate; OA, oxaloacetate; OGC, 2-oxoglutarate carrier; OrC, ornithine/citrulline carrier; PC, pyruvate carrier; PEP, phosphoenolypyruvate; PEPCK, phosphoenolypyruvate carboxykinase; Pyr, pyruvate; UC, urea cycle; UCP2, uncoupling protein 2; 2-OG, 2-oxoglutarate.

For an understanding of the coupling of gluconeogenesis with the urea cycle, it is important to realize that one of two nitrogen atoms in the urea molecule originates from ammonia and the second from aspartate and that the entry of ammonia and aspartate into the urea cycle must be linked. In Figure 3, it is shown that aspartate delivered into the cytosol reacts with citrulline, which contains nitrogen originating from ammonia, to enter the urea cycle in the form of argininosuccinate, which is degraded to arginine and fumarate. Fumarate released from the urea cycle is via malate converted to oxaloacetate, from which gluconeogenesis begins. Therefore, increased flux through the urea cycle, driven by the ammonia in the mitochondria and aspartate in the cytosol, can stimulate gluconeogenesis.

#### 4.1.1. Why Is Alanine a Preferred Substrate for Hepatic Gluconeogenesis?

Alanine likely plays a more important role in hepatic gluconeogenesis when compared with glutamine because the conditions for glutamine conversion to oxaloacetate are not optimal. In the liver, the direction of the flux through GDH1 depends on several factors and, in most conditions, it is directed toward conversion of 2-OG to glutamate (2-OG + NH_3_ + NAD(P)H + H^+^ → Glu + H_2_O + NAD(P)^+^) [38,39]. Therefore, the amount of 2-OG that can enter the CAC to form oxaloacetate is limited. The suggestion is supported by the observation that after administration of L-[^13^N] glutamate into the portal vein, most labels incorporated into aspartate, and a much smaller amount appeared in ammonia [47]. On the other hand, glutamate synthesized by GDH1 can favor the use of alanine for gluconeogenesis by its reaction with oxaloacetate of alanine origin to ensure its transport into the cytosol in the form of aspartate (Figure 3).

Substantial glutamine uptake and alanine release by the small intestine, resulting in increased alanine and decreased glutamine levels in the portal vein that supplies most of the blood to the liver, can also play a role in the preferred use of alanine for hepatic gluconeogenesis [14,48,49,50]. In net balance studies in humans, liver uptake of alanine has been shown to account for ~50% of total amino acid uptake, whereas minimal uptake has been observed with glutamine [42,43].

#### 4.1.2. The Role of Perivenous Hepatocytes

**Figure 4:**
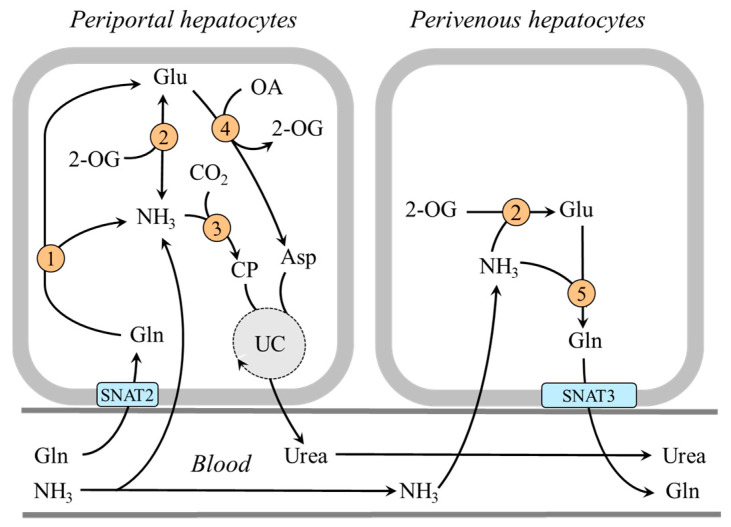
Heterogeneity of periportal and perivenous hepatocytes in glutamine and ammonia metabolism: 1, glutaminase; 2, GDH1; 3, carbamoyl phosphate synthetase; 4, AST; 5, glutamine synthetase. Abbreviations: OA, oxaloacetate; CP, carbamoyl phosphate; SNAT, sodium neutral amino acid transporter; UC, urea cycle; 2-OG, 2-oxoglutarate.

In the central part of the liver lobule (perivenous hepatocytes), GDH1 is not found together with glutaminase but with glutamine synthetase and enables ammonia detoxification to glutamine [39,51,52]. It has been shown that a drop in pH inhibits glutaminase and carbamoyl phosphate synthetase, and subsequent ammonia detoxification into urea in periportal hepatocytes, whereas ammonia detoxification into glutamine and glutamine release into the blood increase in perivenous hepatocytes [37,51,53,54]. Glutamine production by perivenous hepatocytes may help to fulfill the increased needs for glutamine by the kidneys during acidosis and reduce the loss of nitrogen in protein-wasting conditions, like prolonged starvation (Figure 4).

### 4.2. Gluconeogenesis from Glutamine in Kidneys

In the kidneys, gluconeogenesis and glucose release into circulation occurs in the proximal tubules of the renal cortex and accounts for 15–40% of endogenous glucose production in humans in postabsorptive states and increases substantially during starvation and acidosis [7,8,55,56,57]. Human and animal data examining renal arteriovenous differences and the incorporation of gluconeogenic precursors into glucose show that lactate and glutamine are the most important, glycerol less so, and alanine, which the kidneys release into the blood, is the least important [7,8,43,48,58,59].

**Figure 5:**
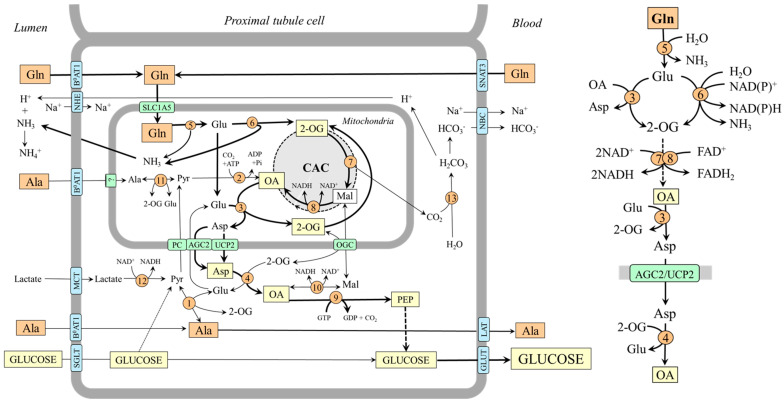
Gluconeogenesis from glutamine and supposed pathways of alanine metabolism in kidneys. The main pathways by which the carbon skeleton of glutamine is converted to oxaloacetate are depicted on the right side. 1, ALT (cytosolic); 2, pyruvate carboxylase; 3, AST (mitochondrial); 4, AST (cytosolic); 5, glutaminase; 6, GDH2; 7, enzymes converting 2-OG to malate; 8, malate dehydrogenase (mitochondrial); 9, PEPCK; 10, malate dehydrogenase (cytosolic); 11, ALT (mitochondrial); 12, lactate dehydrogenase; 13, carbonic anhydrase. Abbreviations: AGC2, aspartate–glutamate carrier 2; B^0^AT1, broad neutral amino acid transporter 1; CAC, citric acid cycle; GLUT, glucose transporter; LAT, large neutral amino acid transporter; Mal, malate; MCT, monocarboxylate transporter; NBC, Na^+^:HCO_3_^−^ cotransporter; NHE, Na^+^/H^+^ exchanger; OA, oxaloacetate; OGC, oxoglutarate carrier; PC, pyruvate carrier; Pyr, pyruvate; SGLT, sodium–glucose-linked transporter; SNAT, sodium neutral amino acid transporter; 2-OG, 2-oxoglutarate.

It has been shown that glutamine accounts for up to 20% of all renal gluconeogenesis in the postabsorptive state, and there is a close relationship with ammonia and bicarbonate ions formation [7,8,60]. Glutamine, which enters the tubular cells by several transporters, including B^0^AT1 on the luminal side and Na^+^/H^+^-dependent system SNAT3 on the basolateral side [61], is rapidly hydrolyzed by the renal type of glutaminase, which is activated when acidity rises [37]. Both glutamate dehydrogenase and aminotransferase pathways are involved in forming 2-OG, which is converted to oxaloacetate by enzymes of the CAC. Under acidosis, because GDH2 is activated by decreased pH, the flux through the glutamate dehydrogenase pathway can be more pronounced (Figure 5).

#### Alanine Metabolism in Kidneys

Unlike the liver, the role of alanine as a substrate for gluconeogenesis seems to be of little significance in the kidneys [48,58]. However, there are several remarkable links between the metabolism of alanine, glutamine, and ammonia. For instance, when plasma alanine is elevated, conversion of this amino acid to ammonia increases, whereas glutamine administration increases renal production of alanine [60,62]. The increase in ammonia production by increased alanine levels is apparently due to alanine transamination with 2-OG to form glutamate, which is subsequently deaminated to liberate ammonia. The rise in alanine production by increased glutamine levels can be explained by the increased supply of glutamate, which acts as a nitrogen donor for alanine synthesis by ALT in the cytosol. Hence, the balance between alanine production and utilization in kidneys is dependent, at least in part, on the concentrations of the reactants. In most conditions, the net balance is towards alanine synthesis [48,58].

### 4.3. Gluconeogenesis from Glutamine in the Small Intestine (Figure 6)

Animal and human studies have demonstrated that the small intestine produces and releases glucose into venous blood in postabsorptive and fasting states, with a high-protein diet, and in diabetes [4,14,15,63,64]. Glutamine is the primary substrate. Enterocytes of the small intestine contain very high glutaminase activities [65,66], and a significant increase in glucose radioactivity has been observed in the mesenteric vein in fasting and diabetic rats infused with L-[U-^14^C]glutamine. In contrast, a very small or insignificant increase was found after infusion of [U-^14^C]lactate, [U-^14^C]alanine, and [2-^13^C]glycerol in fasting rats [64].

Glutamine is transported into enterocytes by B^0^AT1 from the intestinal lumen and several transporters, notably SNAT1, SNAT2, and SNAT4, from the blood [24]. Glutamate produced by glutaminase is converted to oxaloacetate by transamination, not the GDH pathway. That means that 2-OG is formed gradually by glutaminase and AST reactions and then converted through CAC enzymes to oxaloacetate, which contains carbons originating from glutamine. The opinion is based on the relatively low expression of GDH in enterocytes and observations that intestinal glutamine oxidation is inhibited by aminooxyacetate, a known aminotransferase inhibitor [38,67,68].

**Figure 6:**
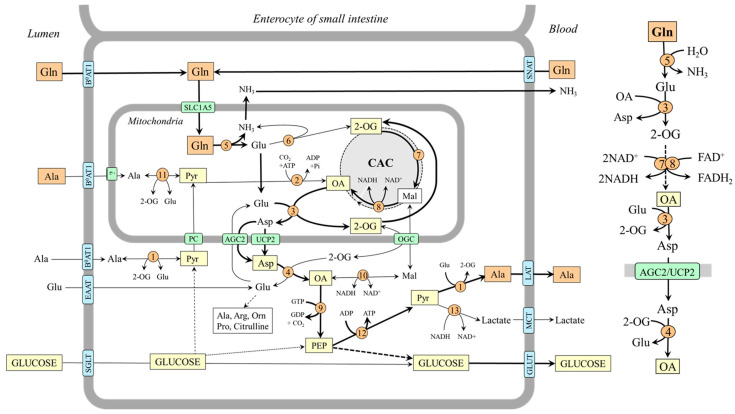
The role of glutamine in gluconeogenesis and alanine synthesis in enterocytes. The main pathway by which the carbon skeleton of glutamine is converted to OA is depicted on the right side. 1, ALT (cytosolic); 2, pyruvate carboxylase; 3, AST (mitochondrial); 4, AST (cytosolic); 5, glutaminase; 6, GDH1; 7, enzymes converting 2-OG to malate; 8, malate dehydrogenase (mitochondrial); 9, PEPCK; 10, malate dehydrogenase (cytosolic); 11, ALT (mitochondrial); 12, pyruvate kinase; 13, lactate dehydrogenase. Abbreviations: AGC2, aspartate–glutamate carrier 2; B^0^AT1, broad neutral amino acid transporter 1; CAC, citric acid cycle; EAAT, excitatory amino acid transporters; GLUT, glucose transporter; LAT, large neutral amino acid transporter; Mal, malate; MCT, monocarboxylate transporter; OA, oxaloacetate; OGC, oxoglutarate carrier; PC, pyruvate carrier; PEP, phosphoenolpyruvate; Pyr, pyruvate; SGLT, sodium–glucose-linked transporter; SNAT, sodium neutral amino acid transporter; 2-OG, 2-oxoglutarate.

#### Alanine and Lactate Production by Enterocytes

The outflow from the small intestine by the mesenteric vein is characterized by decreased concentrations of glutamine and increased concentrations of glucose, ammonia, arginine, citrulline, proline, lactate, and alanine when compared with concentration of these substances in mesenteric artery [14,49,66]. The release of alanine and lactate is undoubtedly important for gluconeogenesis in the liver. However, the pathways of alanine and lactate synthesis in enterocytes are unclear.

The studies using L-[^14^C]glutamine administration have shown that in addition to glucose, glutamine is also a source of pyruvate for alanine and lactate synthesis [64,66]. The glutamine carbons are supposed to appear in pyruvate via phosphoenolpyruvate, which is converted to pyruvate by pyruvate kinase [50]. The ALT and lactate dehydrogenase reactions are driven toward alanine and lactate synthesis, probably due to the low pyruvate carboxylase activity shown in enterocytes of fasted and diabetic rats [64]. LAT2 and LAT4 mediate the efflux of alanine through the basolateral membrane into the blood [24].

## 5. Predicted Roles of Alanine and Glutamine in Gluconeogenesis under Various Physiological and Pathological Conditions

### 5.1. High-Protein Diet

A high-protein diet increases aminoacidemia, particularly BCAA, alanine, and glutamine levels, secretion of both insulin and glucagon, and gluconeogenesis and urea synthesis in the liver [44,45,46,69,70,71]. Alanine and glutamine levels increase more than other amino acids because the increased supply of BCAA (due to negligible intestinal and hepatic BAA catabolism) activates their synthesis in muscles and adipose tissue.

Because most of the glutamine obtained from food is utilized in enterocytes, while alanine is synthesized and transported directly to the liver via the portal vein, alanine seems to be a preferred substrate. The increase in alanine was higher than glutamine in the plasma of rats fed a BCAA- or leucine-enriched diet compared to controls fed by a standard diet [72]. Alanine increased from 527 ± 27 to 667 ± 25 and 627 ± 13 µmol/L (19%) in BCAA and leucine group, respectively; glutamine from 713 ± 25 to 825 ± 12 µmol/L (16%) in BCAA group and 808 ± 19 µmol/L (13%) in leucine group. Evaluation of circadian variations in amino acid levels in men demonstrated marked increase in plasma concentration of alanine after each meal, whereas glutamine varied without the influence of food intake [73].

French investigators have demonstrated that a protein-enriched diet stimulates gluconeogenesis in the small intestine and suggest that increased glucose level in the portal vein initiates signals to the hypothalamic nuclei that contribute to the regulation of food intake [14,74]. Induction of the main gluconeogenic genes has also been shown in the kidneys of rats fed a high-protein diet [75].

### 5.2. High-Fat Diet

Exposure to high-fat diets stimulates gluconeogenesis in the liver [76,77,78,79]. It is assumed that increased production of acetyl-CoA, NADH, and ATP from fatty acid oxidation shifts pyruvate from pyruvate dehydrogenase towards pyruvate carboxylase to form oxaloacetate and decreases fluxes through the CAC and GDH toward 2-OG. Therefore, hepatic gluconeogenesis from alanine is likely activated and glutamine inhibited. The suggestion agrees with data obtained from studies using rat liver perfused by oleate [76]. Furthermore, because high-fat diets contain the necessary amounts of proteins, the supply of alanine to the liver is higher since the majority of glutamine received in food is used by enterocytes to synthesize other amino acids, including alanine.

### 5.3. Early Starvation

Early starvation refers to the first two days of starvation in which the main role in maintaining glycemia has gluconeogenesis from amino acids evidenced by the increased urea release in urine [6,44,46]. The needs of BCAA as a source of nitrogen for alanine and glutamine synthesis are ensured by a decreased ratio of protein synthesis to proteolysis due to the decline in insulin and increase in glucagon concentrations. Alanine synthesis is preferred due to the increased breakdown of glycogen stores in muscles and subsequent pyruvate supply from glycolysis. The glucose synthesized from alanine in the liver can be used in muscles for alanine synthesis again and close the loop known as the glucose-alanine cycle [6].

As a result of the gradual depletion of muscle glycogen stores and subsequent decrease in glycolysis and pyruvate supply, alanine release from muscles decreases, and glutamine production and its potential use for gluconeogenesis increases. Increased use of glutamine for renal and intestinal gluconeogenesis during early starvation has been confirmed by several studies [7,8,14,15,63].

### 5.4. Prolonged (Protein-Sparing) Starvation

After 2–3 days of starvation, the body starts to use fatty acids and ketone bodies as the preferential energy fuel, slowing the breakdown of proteins and urea production. BCAA transamination and their use for alanine and glutamine synthesis in muscles decreases due to impaired 2-OG supply from the CAC inhibited by decreased glycolysis and increased NADH and FADH_2_ supply from fatty acid oxidation. Therefore, plasma BCAA levels temporarily increase, and a marked fall in plasma alanine is observed [56,58,80,81]. A rapid rise in glucose concentration after alanine infusion suggests that the decreased alanine supply from muscles is the main cause of its impaired use for hepatic gluconeogenesis [6].

The shift from glutamine utilization in periportal to its synthesis in perivenous hepatocytes and the simultaneous increase in gluconeogenesis and ammoniagenesis from glutamine in the kidneys compensate for decreased gluconeogenesis from alanine and attenuate the acidotic effect of ketone bodies [58,80]. Cahill showed more than 50 years ago that during prolonged starvation in humans, there is a stoichiometric relation between renal ammoniagenesis and gluconeogenesis, and net renal glucose release contributes approximately to one-half of daily systemic glucose appearance [56].

Unlike short-term starvation, the small intestine’s role appears insignificant due to the reduced glutaminase activity and progressive atrophy of the gut [82,83].

### 5.5. The Final Stage of Starvation

After 7–8 weeks of starvation, energy stores in the form of glycogen and lipids are exhausted, and amino acids become the only source of energy and a substrate for gluconeogenesis. Evidence of the dominant use of amino acids in the terminal phase of starvation is the increase in the activity of enzymes necessary for amino acid oxidation and increased urea synthesis in the liver and its release by the kidney [5,27,28,56].

Increased proteolysis in muscles is associated with the activation of BCKA dehydrogenase and catabolism of the BCAA, which becomes the primary energy substrate and the source of both nitrogen and carbons for glutamine synthesis. Due to the limited pyruvate supply from glycolysis, alanine synthesis in muscles and its use for gluconeogenesis in the liver should be very low.

### 5.6. Acidosis

In acidosis, there is increased extraction of glutamine and activation of ammoniagenesis and gluconeogenesis in the kidneys [84]. Rat renal mRNA levels that encode glutaminase and PEPCK increase 6-fold [85]. In vitro studies performed on cortical renal tubules failed to demonstrate increased glucose production from alanine [86].

In acidosis, the glutamine supply to the kidneys is enhanced because of its activated synthesis in muscles and the liver whereas its hepatosplanchnic uptake is decreased [51,53,87,88,89]. In rats with metabolic acidosis, glutamine release by the hindquarters increased from 79 ± 14 to 391 ± 59 nmol/min/100 g, whereas alanine release decreased from 203 ± 12 to 37 ± 13 nmol/min/100 g of body weight [88]. In the liver, the drop in pH inhibits carbamoyl phosphate synthetase in periportal hepatocytes and ammonia detoxification to urea, whereas ammonia detoxification to glutamine increases in perivenous hepatocytes [51,53]. Markedly reduced hepatosplanchnic glutamine uptake and decreased glutaminase activity in the jejunum have been shown in dogs and rats with acidosis [83,89].

### 5.7. Untreated Diabetes Mellitus of the First Type (T1DM)

In T1DM, the expression of gluconeogenic enzymes is enhanced in all three gluconeogenic organs [14,15,64]. Acidosis resulting from enhanced levels of ketone bodies should increase protein breakdown and glutamine release from muscles and perivenous hepatocytes and its utilization in the kidneys [51,53]. Gluconeogenesis from alanine in the liver is strongly suggested by decreased alanine levels and accelerated hepatic removal of exogenous load of alanine observed in patients with T1DM [90,91,92]. A role in decreased concentrations of alanine in plasma have also impaired pyruvate supply from glycolysis and decreased 2-OG supply for BCAA aminotransferase due to increased fatty acid oxidation in muscles. Therefore, although proteolysis and BCAA oxidation in muscles are increased, decreased alanine and increased BCAA levels are frequent findings in untreated T1DM [92,93].

### 5.8. Diabetes Mellitus Type 2 (T2DM)

Hyperglucagonemia, insulin resistance, and increased fatty acid oxidation are the main causes of increased gluconeogenesis in T2DM [94,95]. Isotopic techniques have demonstrated that the conversion of both alanine and glutamine to glucose is increased in the liver and kidneys, respectively [59,96,97,98,99]. The importance of alanine demonstrates the abundance of mitochondrial isoform of ALT in the liver of animals and humans with T2DM and that silencing of the enzyme attenuates hyperglycemia [99,100].

Similar to T1DM, plasma BCAA levels increase due to impaired BCAA transamination and alanine and glutamine synthesis in muscles [101]. An alternative source of alanine can be glutaminolysis in the small intestine. A source of both alanine and glutamine can be adipose tissue that produces these amino acids in a postprandial state, and its volume is often enlarged in T2DM.

### 5.9. Systemic Inflammatory Response (SIR)

The SIR is a non-specific defense response of the body that occurs in severe illnesses, including sepsis, polytrauma, burns, hemorrhage, and heart failure [102,103]. The cause is a complex of neurohumoral changes, primarily increased levels of cytokines, catecholamines, glucagon, and cortisol. Characteristic manifestations include hyperglycemia, increased gluconeogenesis, insulin resistance, and muscle wasting [104,105]. Increased flux of BCKA through BCKA dehydrogenase activated by proinflammatory cytokines and glucocorticoids plays a role in increased BCAA catabolism that becomes, besides a source of the nitrogen for alanine and glutamine synthesis, an energy substrate and a source of the carbons for glutamine synthesis [29,30,106,107,108,109].

Increased losses of urea by urine indicate that most amino acids released from the muscles are utilized in the liver [110]. In rats with sepsis, marked increases in the net rates of hepatic extraction of both glutamine and alanine with concomitant increases in the release of glucose were demonstrated [111]. Hypoalaninaemia and increased removal of intravenous alanine were shown in patients undergoing total hip replacement [92]. Glutamine utilization by various tissues, particularly the immune system, gut, and proximal tubules of the kidneys, often exceeds its synthesis in muscles, and the glutamine level can drop below 50% of its usual concentration. Such a significant decrease in glutamine is accompanied by an increase in mortality, apparently due to insufficient immune system function [30].

### 5.10. Liver Cirrhosis

Patients with liver cirrhosis have reduced glycogen stores, and their ability to maintain glycemia through hepatic glycogenolysis in a postabsorptive state is limited. Therefore, gluconeogenesis to sustain glycemia is activated much earlier than in a healthy individual [112,113,114,115].

The primary substrate for gluconeogenesis is likely glutamine, whose synthesis is activated by increased ammonia detoxification to glutamine by glutamine synthetase in muscles [116,117,118,119]. Marked increase in glutamate consumption by glutamine synthetase is the main cause of increased flux through BCAA aminotransferase (BCAA + 2-OG → BCKA + Glu), resulting in the decrease in BCAA levels, the drain of 2-OG from the CAC, and impaired mitochondrial function in muscles [120]. The essential role of glutamine in renal gluconeogenesis and glycemia maintenance has been demonstrated by reduced ammoniagenesis in kidneys after overnight glucose infusion in subjects with cirrhosis compared to healthy individuals [121]. The intestine’s role is suggested by observing nearly four times higher intestinal glutaminase activity in cirrhotic patients than in control subjects [122]. Gluconeogenesis from glycerol is not increased in overnight fasted patients with liver cirrhosis [123].

## 6. Summary and Conclusions

The results of in vitro, animal, and human studies demonstrate that both alanine and glutamine play an essential role in gluconeogenesis in various physiological and pathological conditions. Alanine is more important in gluconeogenesis in the liver; glutamine is the primary precursor for gluconeogenesis in the kidneys and small intestine. Because a significant portion of alanine carbons, but not carbons of glutamine, originate from glucose catabolism, glutamine should be recognized as a better substrate for gluconeogenesis than alanine. Furthermore, glutamine’s appearance rate in circulation is higher than that of alanine, and ATP can be produced during glutamine catabolism due to NADH and FADH_2_ release from the CAC during 2-OG conversion to oxaloacetate. On the other hand, alanine conversion to oxaloacetate by pyruvate carboxylase is an ATP-consuming reaction. It can be assumed that the differences in energy requirements for glutamine and alanine conversion to oxaloacetate can influence their entry in gluconeogenesis, especially in states with energy deficits such as cachexia and long-term starvation.

Alanine supply and conversion to oxaloacetate are favored in early starvation and after exposure to high-fat or high-protein diets. Enhanced use of alanine for gluconeogenesis has been reported also in diabetes. In contrast, glutamine use in gluconeogenesis, particularly in kidneys, is stimulated during prolonged starvation, acidosis, illnesses associated with systemic inflammatory response, and liver cirrhosis.

**Figure 7:**
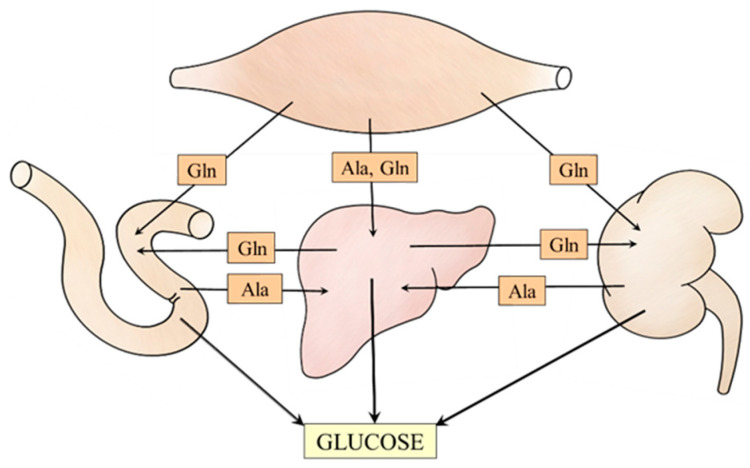
Supposed interactions among muscles, liver, kidneys, and small intestine that ensure alanine and glutamine supply for the needs of gluconeogenesis.

The data discussed in the article suggest meaningful relationships among muscles, liver, kidneys, and small intestine that ensure the supply of alanine and glutamine to individual gluconeogenic organs (Figure 7). For instance, under acidotic conditions, glutamine utilization for gluconeogenesis and urea synthesis in periportal hepatocytes is inhibited, whereas its synthesis in perivenous hepatocytes and release into the bloodstream is stimulated. Subsequently, glutamine is used by kidneys for glucose and ammonia synthesis to maintain glycemia and acid-base balance, respectively.

In conclusion, a better understanding of pathways in which alanine and glutamine, and other gluconeogenic substrates are involved in gluconeogenesis in individual glucogenic organs is required for therapy of disorders of glucose homeostasis.

## Data Availability

Not applicable.
